# Beyond a politics of recrimination: Scandal, ethics and the rehabilitation of violence

**DOI:** 10.1177/1354066116669569

**Published:** 2016-09-28

**Authors:** Jamie M. Johnson

**Affiliations:** University of Sheffield, UK

**Keywords:** Baudrillard, ethics, humanitarian intervention, resistance, violence, war

## Abstract

The practice of contemporary warfare seems to be plagued by scandal. It is often assumed that the act of bearing witness to these moments of ethical failure, in which the relationship between the martial and the ethical breaks down, plays an important role in holding powerful actors to account for their conduct. Considerable faith has been placed in the role of transparency and truth-telling as foundations for normative engagements with war. This article argues that we must be cautious about this investment. Drawing on the work of Jean Baudrillard, this article offers a method for critically reading scandals as a series of line-drawing manoeuvres. Taken together, these manoeuvres demonstrate how scandals function to enable, excuse and obscure the complex landscapes of violence that define the spectacular and mundane sites of contemporary war. Reducing critical engagements with violent practices to a logic of recrimination, scandals often function to revitalise the very principles they appear to contest. Focusing upon the socio-political implications of wartime scandals, this article demonstrates that the performative force of scandals is therefore the reproduction of a violent status quo rather than opening up new spaces for imagining less violent futures. Offering a critical reading of controversies relating to the provision of humanitarian assistance and education in Afghanistan, this article reflects on the ambiguities and anxieties of critiquing violence.

## Introduction

In a time of universal deceit — telling the truth is a revolutionary act. (George Orwell)

War is a controversial enterprise. Recent experiences of liberal or humanitarian warfare have been beset by the revelation of scandals that appear to unsettle and contest the legitimacy of these practices. Exposing these moments of ethical failure, in which the relationship between the martial and the ethical appears to break down, wartime scandals seem to present us with uncomfortable truths about the conduct and legitimacy of military force. Confronted with the disclosure of their wrongdoing, wartime scandals appear to play an important role in holding powerful actors to account by naming and shaming them for practices that would have otherwise remained unaccounted for. Considerable faith has been placed in the normative force of truth-telling and transparency, of which scandals are but one form, for providing a platform for contesting the legitimacy of dominant wartime narratives and practices.^1^ It is through this dominant cultural understanding of the legitimacy and necessity of tireless truth-seekers that we can understand the socio-political significance of actors as varied as Wikileaks, the Bureau for Investigative Journalism and the Chilcot Inquiry. Understood as a basis for critique and popular resistance, scandals are often interpreted as a revolutionary act of bearing witness, in which *truth is spoken to power*.

This article does not dispute the idea that scandals are acts of truth-telling. It does, however, contest and complicate how we should understand what it means to speak truth. This is motivated by a concern with the socio-political function of wartime scandals. An important distinction is therefore made between two ways of understanding the act of truth-telling. Understood in terms of *speaking truth to power*, truth-telling is a process of exposing a transgressive reality that exists independently of its disclosure and denunciation. This article, by contrast, focuses on the notion of truth-telling in terms of how it demonstrates the *power of truth*. The emphasis of such an approach shifts from focusing upon what scandals make visible, that is, how they reflect an independent and prior reality, and instead urges us to understand scandals in terms of what they make possible. From this perspective, scandals can be seen as crucial sites in the reproduction of particular representational logics and knowledge claims. They can also be seen as enabling and realising a particular form of politics.

This politics is not the one we would perhaps expect. The argument of this article is that scandals reduce critical interventions to a logic of recrimination in which acts of excessive violence are denounced for ‘not following the rules of the game’ (Baudrillard, 1994: 15). Confined to such a logic, scandals may, in fact, serve to blunt the possibilities of critically reflecting upon particular wartime practices. Perversely, far from a demonstration of the fragility of the relationship between the martial and the ethical, this logic of interpreting and responding to ethical failure through scandal serves to revitalise the very practices it appears to call into question. To be clear, the argument presented here is that through wartime scandals, the legitimacy of violence is secured and reaffirmed rather than contested. Moments of ethical failure are therefore, counter-intuitively, the conditions of possibility for *more* rather than *less* violence. Far from being a revolutionary act, the underlying logic of wartime scandals is to maintain a violent status quo. The act of truth-telling is borne out of the desire to end impunity in order to ensure that there are consequences for particular forms of action. The argument of this article is that the act of truth-telling has its own consequences, which require greater scrutiny.

This argument will proceed in two parts. Inspired by the work of Jean Baudrillard, the first part outlines a method for interpreting scandals as a series of line-drawing manoeuvres. Taken together, these manoeuvres demonstrate how scandals are not a neutral moment of exposure in which the terms and meaning of the scandal are established by the character of the act itself. Instead, accountability through scandals relies upon a grammatical logic of accountancy in which certain events are held to account in particular ways, while others remain unaccounted for. Scandals are therefore a political form of problematisation in which acts are interpreted and responded to through a simultaneous logic of inclusion and exclusion. Rather than understanding scandals as moments that render acts transparent to scrutiny, the first section will instead argue that we must recognise them as constructed sites of *hyper*-visibility and *un*-visibility in which certain acts emerge through a process of narration. The second part will demonstrate that the function of this storytelling is to enable, excuse and obscure the complex landscapes of violence that define the spectacular and mundane spaces of the conflict in Afghanistan. This will be achieved through a critical reading of two scandals relating to the delivery of humanitarian relief and textbooks within Afghanistan.

## Simulating scandal and the politics of recrimination

*Watergate is not a scandal*: this is what must be said at all costs. (Baudrillard, 1983: 28, emphasis in original)

Existing post-structural analyses of war and violence have drawn significantly from the work of Michel Foucault (Dillon and Neal, 2008; Dillon and Reid, 2009),^2^ Jacques Derrida (O’Callaghan, 2012), Judith Butler (Gregory, 2012; Zehfuss, 2009), Paul Virilio (Der Derian, 2009) and Gilles Deleuze (Evans and Reid, 2013; Reid, 2003). In spite of his infamous claim that the Gulf War *will not take place/is not taking place/did not take place* (Baudrillard, 1995), the work of Jean Baudrillard has been surprisingly marginal to much of this scholarship. More recently, this provocative series of essays has inspired a growing literature on the ontology, aesthetics and phenomenology of contemporary warfare (Artrip and Debrix, 2014; Nordin and Öberg, 2015; Van Veeren, 2011). What remains lacking within this literature, however, is a sustained reflection on the important contribution that Baudrillard’s thinking can make to our understanding of the relationship between war and ethics. Specifically, Baudrillard offers us important insights into the character, reproduction and socio-political function of ethical arguments about war.

Unlocking this contribution requires a departure from Baudrillard’s writing on the Gulf War. Instead, we must turn to his earlier writing on simulacra and simulation (Baudrillard, 1983, 1994). It is here that Baudrillard articulates a lesser-known incitement to his readers. Specifically, he claims that Watergate, the scandal *par excellence* that has bequeathed to all subsequent scandals the suffix ‘-gate’, was not and must not be understood as a scandal. Instead, he argues that Watergate is a simulation of scandal, which functions to reproduce the very morality principle that appears to be distressed by the apparent exposure of a transgressive act.

This section will now unpack the meaning and ethico-political significance of Baudrillard’s position and demonstrate its implications for thinking about the limits and possibilities for constructing ethical arguments about war. Rather than providing a detailed explication of Baudrillard’s wider thinking about hyper-reality, which is beyond the scope and purpose of this article, the article will instead explore the significance of Baudrillard’s thinking through the metaphor of line-drawing. Understanding scandals as a series of line-drawing manoeuvres (Pin-Fat, 2010), each of which plays a crucial role in shaping ethical imaginaries about war, allows us to develop Baudrillard’s thinking into a critical method and ethico-political strategy for exploring and contesting the ethics of war.

### First manoeuvre: Crossing lines

Watergate above all succeeded in imposing the idea that Watergate *was* a scandal. (Baudrillard, 1983: 27, emphasis in original)

For Baudrillard, the first achievement of a scandal is to secure the idea that there is a scandal. The denunciation of a particular act as scandalous relies upon the identification of transgression, of a *line having been crossed*. This relies upon a popular acceptance that such a line is real and that it carries moral significance. For a scandal to resonate, it must succeed in plausibly demonstrating that an act has overstepped a line that marks the boundary between permissible and impermissible forms of conduct. A scandalous act is therefore understood as a form of behaviour that has either intentionally or unintentionally gone awry and finds itself crossing over into the transgressive space of the illicit, the taboo and the forbidden. Understood as such, scandals are interpreted as a failure of navigation, a form of ethical trespassing as a result of either an inability or unwillingness to be directed by a prescribed moral compass, which guides us on how to remain on the right side of the line.

The first line-drawing manoeuvre of the scandal is therefore to highlight which line has been crossed and to assert that this line is drawn deeply, by which we mean that it has a depth of meaning, or what Véronique Pin-Fat (2010: 125) refers to as ‘ontological depth’. Securing the stability of this position allows for judgement and the confident projection of outrage at a particular form of conduct. It is from this position that a movement can be made from transgression towards resolution. This first manoeuvre offers us a number of important insights about the ethical character of war.

First, there is an assumption, betrayed by the performance of wartime scandals, that war has — or at least should have — values and therefore has value. After all, you cannot have scandal without some understanding of, and association to, a set of cherished principles or acceptable form of conduct. The first moment of revelation in a scandal is not therefore the particular dimensions of the transgressive act. Before even this, the occurrence of scandal indicates the existence of a normative architecture that allows for ethical judgements to be made about conduct in war. Wartime scandals therefore rely upon the assumption that war can and should be ethical. The identification of failures in the ethicality of wartime conduct therefore assumes the possibility of success. If wartime conduct can fail, then presumably it can succeed. This is the first lesson to be taken from the scandal. The occurrence of wartime scandals therefore tells us that conduct in war is exposed to forms of ethical scrutiny that govern the prosecution of violence.

Second, while the existence of such lines tells us that conduct in war is regulated by ethical judgements, the ubiquity of scandal across almost every dimension of the prosecution of the ‘war on terror’ is indicative of the breadth and depth of contemporary humanitarian or liberal warfare’s moralising quality. The frequency of scandal therefore not only tells us that such lines exist, but also reflects the multiplicity of lines that govern contemporary warfare. Indeed, what makes war so seductive as an instrument of foreign policy is its apparent compatibility with a range of moral principles that attempt to delineate the permissible from the impermissible in relation to the grounds for going to war (the *jus ad bellum*), conduct within war (the *jus in bello*) and increasingly the termination of conflict (the *jus post bellum*).

While wartime scandals are by no means novel historical developments, the practice of humanitarian or liberal warfare is increasingly punctuated by the revelation of transgressive events. This observation is testament to the intensification of the relationship between the martial and the ethical. Specifically, it shows us the increasingly important role that ethical judgements play in securing the legitimacy of contemporary conflicts, to the point that humanitarian or liberal warfare ‘is no longer just a form of war but has become virtually synonymous with permissible war itself’ (Lawler, 2003: 151; see also Dexter, 2007; 2008). The propensity for scandal does not therefore indicate a widespread dissatisfaction with the idea that violence can be ethical. Instead, this tendency to scandalise particular acts of transgressive or *excessive* violence can be seen as an indication of the strength of conviction in this relationship.

Third, the location of where each of these lines has been drawn gives us an insight into the specific ways in which we differentiate between permissible and impermissible forms of wartime conduct. The denouncement of civilian casualties, for example, demonstrates the ethical significance of the principles of discrimination and non-combatant immunity to the legitimacy of contemporary liberal warfare. It is not enough, however, to say that scandals reveal the specific principles that govern contemporary war. Existing theories of just war and juridical frameworks regarding the laws of armed conflict can perform such a function by offering formally codified and widely accepted foundations for such distinctions to be drawn. What distinguishes this approach is that the meaning of a scandal is not simply determined by the principle that has been transgressed, by the specific *line that has been crossed*. The revelation of scandal is not a neat or neutral epistemological process of exposure and disclosure in which acts are uniformly judged in accordance with an ethical grammar that distinguishes the permissible from the impermissible.

The meaning and logic of a scandal exceeds these strictly ethical and juridical frameworks. Instead, transgressions are interpreted through and implicated in the reproduction of wider frameworks of intelligibility within war. Consider, for example, the revelation of ‘prisoner abuse’ within the Abu Ghraib detention facility. While this scandal clearly invoked the idea that these violences were a departure from the rule of law and widely accepted norms regarding the humane treatment of prisoners, this is not the only way in which these events were rendered intelligible. This moment was not simply read as an instance of violence that overstepped the line in a strictly juridical sense. As Melanie Richter-Montpetit (2007: 38) has argued, these violences were also interpreted through a ‘pre-constructed, heterosexed, racialised and gendered script’. The transgressive dimensions of ‘prisoner abuse’ in Abu Ghraib were not therefore solely, or perhaps even primarily, understood in relation to a set of codified ethical principles that were violated. The signification of these violences as scandalous also drew upon and reproduced a wider set of representational logics. As such, the dominant narrative of the violences at Abu Ghraib became an individuated story of ‘womanhood or sexuality gone awry’ (Sjoberg and Gentry, 2007: 70). This fetishisation and denouncement of a ‘few bad apples’ serves to obscure a more systematic insight into the widespread use of extra-juridical and extra-territorial rendition, torture and killing that has defined the ‘war on terror’.^3^

Unpacking the first line-drawing manoeuvre in this way allows us to understand that scandals are not detached and dispassionate arbiters of ethical conduct. As opposed to approaches that focus upon adjudicating wartime conduct in terms of its adherence or deviation from standards and thresholds defined by pre-given ethical frameworks, this approach to the ethics of war draws attention to an *everyday ethical vernacular* (see Bubandt, 2005; Vaughan-Williams and Stevens, 2016): a diffuse, decentred and circulating discursive economy through which particular acts of violence are rendered intelligible. It is a way of thinking about ethical arguments about war that focuses less on how particular acts of violence are problematised in relation to defined and fixed norms. The process is less clearly determined. To understand the scripting of ethical failure, of scandalous transgressions, we must therefore come to understand the complex intersections and resonances between ethical imaginaries and other representational logics.

It is therefore important to understand scandals as primarily political rather than epistemological events. The exposure of a scandal is not simply a process of correctly naming an event as such. Rather, scandals are constructed sites of *hyper-*visibility that exceed the ethical frameworks that they invoke.^4^ Such an understanding of scandals leads us away from the idea that they are, by exposing and disclosing hidden transgressions, a means of *speaking truth to power*. Instead, understanding this first line-drawing manoeuvre begins to demonstrate to us how scandals are a manifestation of the *power of truth*. Such an approach to scandals draws us away from an idea that they are determined by the intrinsic qualities of the act itself and instead urges us to reflect on the socio-political function of this process of signification.

### Second manoeuvre: Redrawing lines

The denunciation of scandal always pays homage to the law. (Baudrillard, 1983: 27)

The first line-drawing manoeuvre of a scandal allows us to note that a *line has been crossed*. As has been shown, the observation of transgression therefore offers us privileged insights into the existence, prevalence and location of these lines. By tracing these crossed lines, we gain an understanding of the everyday ethical vernaculars that reflect popular understandings of the relationship between war and ethics. The danger of understanding this first manoeuvre alone is that it largely leaves intact the heroic notion of scandals as a means of revealing ethical transgressions. The observation that the signification of scandals exceeds the ethical norms that they invoke could simply be read as a suggestion that to understand the scripting of ethical failure, we must understand how these events are embedded within broader representational regimes. Understood as such, scandals simply reflect the complex and contingent resonances between ethical imaginaries and other dominant discourses and stories. In this sense, the first manoeuvre is not really involved in line-*drawing* at all; it is simply observing that lines have been drawn.

Taken on its own, what this manoeuvre gestures towards but fails to account for is the performative force of scandal: how the invoking of particular lines ‘produces the effect that it names’ (Butler, 2011: xii). We must therefore supplement this first manoeuvre with a second in which scandals are not simply read as the *crossing of a line* that exists independently of this apparent observation. Instead, scandals must be understood as a process of *redrawing the line* that has been transgressed. In this sense, the first and second manoeuvres are not really separate manoeuvres at all. Scandals do more than simply reference norms and principles; they are productive of them. The second manoeuvre points to how norms come to be revitalised and pursued with renewed vigour; it allows us to understand the constitutive function of the first manoeuvre. To be clear, the performative force of scandal is to regenerate the very principles that are distressed by their apparent transgression. Ultimately, this is the success of ethical failure.

Scandals, and ethical engagements with war more generally, must be understood in terms of their ‘socio-political effects [which] impact on our collective understanding of war itself’ (Dauphinée, 2008: 50). The second manoeuvre draws our attention to a particular dimension of this effect. Specifically, it demonstrates the way in which scandals function as what Baudrillard (1994: 18) refers to as an example of ‘operational negativity’: a securing of a positive reality through the denouncement of its inversion, subversion or semblance. To help elaborate on this function, Baudrillard considers the doctrine of iconoclasm. The iconoclastic argument forbids the worshipping of images of the divine on the basis that ‘the divinity that breathes life into nature cannot be represented’ (Baudrillard, 1983: 7). What underpins iconoclasm is the assumption that there is a divine presence against which particular representations can be judged; there has to be a presence that allows for the identification of its absence.

For Baudrillard (1983: 11), the denouncement of various signs as false representations of the real ‘masks the absence of a basic reality’. In this sense, God is not simply dead; rather, God never existed, and there has only ever been the simulation of a divine presence. The notion of operational negativity therefore offers Baudrillard a means to develop his wider thinking about simulation and the hyper-reality of the symbolic order through which social reality is constituted. While this potentially opens up interesting avenues regarding the ontological status of the ethical architecture of war, understanding the second manoeuvre requires us to explore a different dimension of the socio-political function of this logic.

As has been shown, iconoclasm performs an important pedagogic role. If God cannot be represented, then God surely exists: this is the underlying message of the iconoclasts. Operational negativity highlights an absence in order to affirm the veracity of an invoked presence. However, this denouncement does more than reaffirm an underlying reality principle. It also performs a crucial regulatory function. The force of this denunciatory logic is to police conduct in accordance with the transgressed law: *you shall not make for yourself a carved image, or any likeness of anything that is in heaven above*. Denunciation therefore attempts to resolve transgression by demanding conformity to a cherished principle or commandment. The effect of identifying deviation is to ensure a return to the norm. The tendency of denunciation is towards a *re*-solution, usually understood as a securing and reproduction of the status quo. Denunciation therefore performs a conjoined pedagogic-regulatory function in attempting to secure both the *power of truth* and the *truth of power* (Dillon, 2015). It is in both of these senses that we must understand Baudrillard’s (1983: 27) claim that: ‘The denunciation of scandal always pays homage to the law.’

Scandals, as an example of operational negativity, are therefore not necessarily moments through which particular principles come to be scrutinised or disputed. Instead, the function of operational negativity is often to:

regenerate a moribund principle through simulated scandal, phantasm, and murder — a sort of hormonal treatment through negativity and crisis. It is always a question of proving the real through the imaginary, proving truth through scandal, proving the law through transgression … Everything is metamorphosed into its opposite to perpetuate itself in its expurgated form. … Power can stage its own murder to rediscover a glimmer of existence and legitimacy. (Baudrillard, 1994: 18–19)

From this reading, scandals do not emerge as a space for contesting or rethinking the legitimacy of a particular social order. Instead, what appears to be a moment of disruptive failure is actually crucial to the rehabilitation and regeneration of the very social order that appears to have failed. What is troubling from the perspective of this second manoeuvre is how critical arguments about the ethics of war become implicated in the very practices that they appear to challenge. Understanding this complicity in the conditions of possibility of military violence requires us to understand the ways in which scandals shape the possibilities and limits of critical responses to perceived ethical failures in wartime conduct.

Baudrillard’s concern with thinking within the logics of the scandal is that it reduces critical thought to a logic of recrimination. Scandals present a simple decision in the face of an event: ‘to receive it as rational *or* to combat it in the name of rationality, to receive it as moral *or* to combat it in the name of morality’ (Baudrillard, 1994: 15, emphasis in original). It is these grammatical terms of the scandal that are particularly problematic as, through them, critical thought becomes confined to performing a regulatory function in support of the logics of a particular morality or rationality. To denounce a particular act ‘for not following the rules of the game’ accepts and affirms that if only these rules were followed, then a particular form of behaviour would be unproblematic. This account of critique as recrimination blunts the possibilities of critical thought, largely confining it to a logic of problem-solving whereby the ethical problem of war is reduced to the identification — through transgression — and *re-*solution of a series of problems through a return to the norm. Problematically, this not only leaves unquestioned and untroubled the norm that it invokes, but actively serves it as, understood in this way, the possibility of transgression implies that *if transgression were eliminated, war would be a wholly moral exercise*.

Far from undermining the possibilities for war by exposing its apparent failures, scandals are involved in the production and reproduction of the very principles upon which contemporary warfare is made possible. This is the success of ethical failure. In short, the durability of the understanding of war as a legitimate enterprise comes to rely, in part, upon the managed exposure of its fragility. Ethical failure in warfare is therefore crucial to upholding the very principles that make violence possible. Perversely, no matter how well intentioned, scandals are complicit in a virtuous cycle that reproduces the legitimacy of virtuous war. In this sense, ethical failure comes to affirm and necessitate more *successful* forms of violence.

Recriminations against the perceived breakdown of the relationship between the martial and the ethical are in danger of confining critiques of wartime violence to the process of policing conduct in war against a series of fixed standards and thresholds. It is in this sense that we should understand scandals as a watchdog on government; not as speakers of *truth to power*, but rather as speakers of the *power of truth*. Far from challenging the construction of war as an instrument of ethical foreign policy, the terms of critical engagement are such that opposition to particular forms of wartime conduct becomes implicated in the reproduction of the very thing that it may set out to challenge or dismantle.

Of course, not all responses to scandals are motivated by this desire. For example, many responses must be situated within wider pedagogic efforts designed to learn from and improve the efficacy and ethicality of wartime conduct. Viewed from the perspective of this ‘fail again, fail better’ approach, scandals are a window of opportunity to refine rather than refute the terms of ongoing violence. The danger and tragedy of scandal is that it is hard to conceive of ways of critiquing war that escape this logic. Rather than creating spaces for imagining less violent futures, scandal overwhelmingly tends towards a politics of recrimination and the resolution of largely individuated moments of ethical failure through technical fixes. The problem of scandal is therefore that it threatens to make iconoclasts of us all: urging us to denounce and combat false or aberrant forms of violence in the name of a purer and truer form of violence that we are urged to pursue with a renewed zeal and vigour.

### Third manoeuvre: Boundary lines

Watergate. Same scenario as Disneyland (an imaginary effect concealing that reality no more exists outside than inside the bounds of the artificial perimeter). (Baudrillard, 1983: 26)

It has been argued that the denouncement of transgression, of a *line being crossed*, must be understood as a simultaneous moment of its *redrawing*. What emerged from this was an understanding of scandal as a means of holding certain acts of violence to account. The second line-drawing manoeuvre emphasises a particular dimension of the socio-political function of this logic. Specifically, it demonstrates how scandals confine critical reflections on war to a form of moral accountancy, the performative force of which is to secure and revitalise the very principle that had been distressed by its apparent transgression.

As a form of moral accountancy, scandals are a moment of *hyper-*visibility in which certain acts of violence are interpreted and responded to according to what has been referred to as an everyday ethical vernacular. The third line-drawing manoeuvre aims to supplement this reflection on the ways in which we think and speak about wartime scandals with a focus on the limits of our anxieties about violence. In a sense, scandals are a process of managing and containing anxiety. Whereas the second manoeuvre unpacks the palliative function of scandals in terms of resolving anxiety, an expurgation that allows the transition from anxiety to a form of moral tranquillity, the third manoeuvre traces the boundary-producing performance of a scandal in order to unsettle wider categories of comfortable violence that are implicitly invoked through wartime scandals. This final manoeuvre therefore asks us to consider those forms of violence that are left unaccountable through the problematisation of other violences as scandalous. It is a means of coming to terms with the discomforting remainders that are enabled, obscured and excused through the process of moral accountancy that underpins scandals.

Scandals function through the drawing of a neat perimeter around a particular site within which transgressive events have occurred, and from which the lessons of a scandal are to be learned. Inside this perimeter, such as that drawn around the complex of the Watergate hotel, is a *hyper-*visible site of extraordinary political toxicity (Baudrillard, 1983: 27). Scandals implore us to understand that it is in relation to the events that occurred within this space that our outrage should be targeted. The logic of this manoeuvre is to assure us that beyond this perimeter, the wider moral architecture remains intact and that this architecture is moral. Watergate was a scandal because it was the failure of the liberal-democratic capitalist order within a particular site; the order itself is not only innocent, but, as has been argued, comes to be revivified through being held up as the standard against which conduct has fallen. The perimeter of scandals therefore functions to delineate between a realm of disruptive moral panic and the tranquillity of the legitimate status quo. Scandals do not simply function to purify toxic spaces; they also assure us of the wider morality of a particular practice or social order.

Consider, for example, the notion of a war crime. The understanding of the war crime as a distinct form of violence is premised upon the idea that you can commit a crime *against* war. War crimes can only be rendered intelligible in relation to a normative architecture that functions as a regulative ideal against which certain violences are deemed to lack the requisite necessity, proportionality or humanity. The result of singling out the illegality of certain acts of *excessive* violence is to offer all other acts of war the appearance of legitimacy. Pathologising particular instances of violence as ‘uniquely abhorrent’ normalises those acts of violence that are not deemed to be criminal (Dauphinée, 2008: 50). It is in this sense that Elizabeth Dauphinée (2008: 55) argues that war crimes trials play a crucial role in ‘securing the legitimacy of war itself’. The war crimes trial functions to make possible another form of violence. Often framed as a proportional and necessary response to the excesses of that which is scandalous, we should be concerned about the ways in which ethical failure functions to demand more, albeit *better*, not less violence.

The staging of ethical failure, in the form of terrorist atrocities (Dexter, 2012) or human rights violations (Kennedy, 2012), is therefore central to many contemporary arguments about the legitimacy of humanitarian or liberal warfare. The *hyper-*visibility of particular violences is always simultaneously marked by the *un*-visibility of other forms of violence, which remain largely unaccounted for beyond the perimeters of moral outrage. Wartime scandals establish a hierarchy of death and injury in which particular bodies are highlighted as problematic, while others are marginalised and silenced beyond the limits of our anxiety and outrage. The third manoeuvre allows us to scrutinise this boundary between permissible and impermissible death and injury in warfare. Mindful of the dangers highlighted in the second manoeuvre, we must be careful that we do not respond to this by simply re-scandalising those forms of death and injury that currently reside beyond the perimeters of the exceptional. While it is challenging to avoid this tendency to scandalise that which is undesirable, we must be wary of the limits that this places upon the possibilities of critical thought. Rather than contesting the boundaries of the permissible and the impermissible by drawing new lines, it is preferable to recognise that such lines are already artificial, blurred and leaky, and that the exception haunts the mundane just as the mundane haunts the exception.

## Critically reading scandals

The first part of this article has outlined a method for critically reading scandals as a series of line-drawing manoeuvres. This section will now apply this method to two scandals that emerged during the early stages of the ongoing conflict in Afghanistan. The first relates to a controversy regarding the delivery of humanitarian relief, in which food packages were confused with unexploded cluster bomblets. This reading will focus upon the *hyper-*visibility of scandals, thereby allowing us to explore the first two manoeuvres. The second relates to the gradual unfolding of revelations about the provision of textbooks that sought to promote a violent and radicalised student-subject. By contrast, this reading will focus upon the third manoeuvre, demonstrating the *un-*visibility of scandals in order to recover the discomforting remainders from this process of moral accountancy. Taken together, these readings demonstrate the character, productiveness and limits of collective understandings of violence.

### Life and death from above

As we strike military targets, we will also drop food. (George W. Bush, 2001)

In late October 2001, CNN (2001) reported an incident in Khoja Bahauddin, a village in the Takhar province of north-east Afghanistan, in which:

Terrified villagers said they ran out of their mud huts when they heard loud thumps on their roofs. ‘We all woke up at two in the morning and thought we were being bombed,’ said Sharaf Ullah. ‘But when we looked around we found packets of food.’

The article describes the surprise and delight of the villagers at receiving Humanitarian Daily Rations (HDRs). These rations, which are enclosed in bright yellow packaging, carried the message: ‘A food gift from the people of the United States of America’. Unfortunately, the confusion experienced by these villagers, in distinguishing a gift from a weapon, was to be repeated across the country with lethal effect as HDRs were not the only yellow containers to fall from the skies over Afghanistan. As well as HDRs, the coalition forces were also dropping cluster bombs. With the yellow unexploded cluster bomblets bearing a strong resemblance to the HDRs, the line between life-saving and life-ending airdrops was seriously complicated for the people of Afghanistan.

Within weeks, stories began to emerge highlighting the tragic consequences of this confusion. In late November, BBC News (Muir, 2001) reported the story of Sayyid Ahmad Sanef, a 15-year-old boy from Western Pakistan, who ‘spotted what he thought was one of the yellow food packets. He picked it up to look at it. It blew his head off.’ In a speech to the House of Representatives, Congresswoman Cynthia McKinney (2001: H7392) argued that while the distinction between the two was far from apparent, it was of deadly significance:

One is life and the other is death. The squarish one is the food. The roundish one is a cluster bomb. [N]ot only do innocent Afghans have to worry about the Taliban, not only do they have to worry about landmines left over from the last war, not only do they have to worry about starving to death and the approaching winter, now they have to worry about bombs that look like food. I think I have heard it all now, Mr Speaker.

Emerging from McKinney’s speech is a particular logic of problematisation that establishes the terms of the scandal. Specifically, the scandal revolves around the transgressive resemblance of instruments of violence with instruments of compassion. It is that bombs look like food, that life is indistinguishable from death. The visual indeterminacy of these two containers is interpreted as marking a transgressive, albeit unintentional, conflation of the dual imperatives of the conflict in Afghanistan.

From its inception, the conflict was tasked with a joint biopolitical function: to extend compassion to the people of Afghanistan, who coalition forces were there to *make live*, while simultaneously extending violent force to the Taliban, who coalition forces were to *let die* (Dillon and Reid, 2009). This coupling of sustenance and violence is an increasingly necessary component in the legitimisation of contemporary conflicts. Breaching the cherished principle of non-combatant immunity, which demands that for military force to be legitimate, it must discriminate between combatants and non-combatants, this identification of transgression highlights the centrality of civilian protection to contemporary ethical imaginaries.

This has not always been the case (Colonomos, 2007; Zehfuss, 2011). The denouncement of civilian casualties in Afghanistan as contrary to the desired conduct of coalition forces demonstrates that contemporary conflicts are fought according to a transformed set of norms and preferences regarding the permissibility of particular forms of death and injury. This transformation is clearly apparent in how prominent members of the military leadership have articulated their understandings of the legitimate means and aims of armed conflict. Consider, for example, General Curtis ‘Bombs Away’ LeMay, one of the architects of Allied strategic air power during the Second World War. When asked to formulate his understanding of the end goal of war, LeMay stated simply: ‘You have got to kill people, and when you have killed enough of them they stop fighting’ (cited in Scheuer, 2004: 85–86). Speaking in the aftermath of the Pacific campaign on the occurrence of civilian casualties, LeMay refuses to acknowledge the ethical significance of this category, remarking that ‘[t]here are no innocent civilians. It is their government and you are fighting a people, you are not trying to fight an armed force anymore. So it doesn’t bother me so much to be killing the so-called innocent bystanders’ (cited in Sherry, 1987: 287).

Such an understanding of what constitutes the legitimate aims and means of military operations, representative of the Kubrick-esque caricature of military leadership as ‘cigar-chomping, knuckle dragging Neanderthals, itching to bomb yet another poor Third World nation’ (Campbell, 1998: 357–358), seems far-removed from the more strategically nuanced and empathetic military leadership in Afghanistan. Within this context, the emphasis of military operations is primarily focused upon achieving *reconstructive* rather than *destructive* objectives. It is in this sense that Admiral Mike Mullen (cited in CNN, 2008) asserted that ‘we can’t kill our way to victory’. Instead, what becomes apparent is how the conflict in Afghanistan was understood and legitimated primarily as a fight *for* the people of Afghanistan, rather than *against* them. For example, speaking of his role in counterinsurgency operations in Afghanistan, General Stanley McChrystal (cited in Rose, 2013) explained that the ‘whole point of war is to take care of people, not just to kill them. You have to have a positive reason that protects people, or its wrong.’

The varying permissibility of civilian casualties demonstrates a transformation in popular understandings of what constitutes legitimate warfare. While the drawing of lines is by no means unique to contemporary warfare, scandals allow us an important opportunity to trace the historic evolution of norms regarding the permissibility of specific forms of warfare. Wartime scandals must therefore always be understood in terms of their historicity. To be clear, the conditions of possibility for scandal are the contingent constellation of dominant cultural understandings of where particular lines have been drawn and how they resonate with wider representational logics, which, taken together, constitute the *everyday ethical vernaculars* that define the character and discourse of war. It is in this sense that we must understand the scandal relating to HDRs.

What emerges from this scandal is what Slavoj Žižek (2002: 94) refers to as a ‘coincidence of opposites’, in which:

military action against the Taliban is almost presented as a means to guarantee the safe delivery of humanitarian aid. We thus no longer have the opposition between war and humanitarian aid: the two are closely connected; the same intervention can function on two levels simultaneously: the toppling of the Taliban regime is presented as part of a strategy to help the Afghan people oppressed by the Taliban.

For contemporary military operations to be legitimate, they must not only be accompanied by humanitarian operations, but be largely subordinated to them. Violence has therefore come to be repackaged in the service of humanitarian outcomes. It is important to clarify that this ‘coincidence of opposites’ does not imply their equivalence; simultaneity does not imply synonymy. This intimacy between the technologies of war and peace has its limits and they are strongly policed. McKinney’s speech demonstrates that a line must be maintained between these two endeavours and that any leakages are to be interpreted as scandalous transgressions of a cherished principle. To be clear, the line that is being drawn by this scandal is that instruments of war must be accompanied by and serve instruments of peace. Importantly, though, these instruments are, and must remain, distinct: ‘one is life and the other is death’ (McKinney, 2001: H7392).

The denouncement of this apparent transgression, of a *line being crossed*, therefore offers us an insight into the character of the normative architecture that regulates contemporary conflict. As was argued in the first section, denouncement invokes what has been referred to as an everyday ethical vernacular that exceeds these principles. Demonstrating the wider representational practices through which these violences are rendered intelligible allows us to explore the socio-political function of this scandal. Put simply, it allows us to note how the denunciation of a *line being crossed* is also, simultaneously, its *redrawing*.

To help elaborate this point, it is important to note that coalition forces were not the only actors denounced for their responsibility in causing civilian casualties. As indeterminate yellow containers fell from the skies, the Taliban were also being called to account for harms caused to civilians. While invoking the same cherished principle, that is, non-combatant immunity, significant differences emerge between the denunciation of these actors’ involvement in civilian casualties. A comparison of these expressions of outrage allows us to demonstrate how the interpretation of scandal exceeds the principles they invoke, and is instead told through what has been referred to as everyday ethical vernaculars.

While the denunciation of coalition civilian casualties presents these moments as the *excess* of humanitarian violence, those caused by the Taliban are interpreted as an apparent demonstration of the basic character and purpose of their violence. Both are taken to be testament to the moral character of the actor who perpetrated the violence. However, while the former is a lapse in the conduct of an otherwise well-intentioned actor, the latter is an indicator of their cruelty and barbarity. Terminology plays a crucial role in entrenching this distinction. Coalition civilian casualties (or ‘CIVCAS’) are referred to as ‘accidents’ and ‘collateral damage’, while those committed by the Taliban are denounced as ‘acts of terrorism’ and ‘war crimes’. While the coalition is decried for having fallen below the standards to which they aspire, the Taliban are accused of the deliberate subversion of them.

Such a logic was articulated by Barack Obama (2009) during his Nobel Peace Prize acceptance speech, in which he argued that:

as we confront a vicious adversary that abides by no rules, I believe the United States of America must remain a standard bearer in the conduct of war. That is what makes us different from those whom we fight. That is a source of our strength.

While both instances of civilian casualties result in a rule being broken, a crucial distinction is made between rule-*followers* and rule-*breakers*. The former respect and attempt to adhere to humane and humanitarian standards of proportionate and necessary violence; the latter deliberately and wantonly ignore them. What this demonstrates is that the interpretation of ethical failure is intimately bound up with the production and reproduction of a wider politics of identity that informs everyday ethical vernaculars. The ability to narrate and define scandals in particular ways demonstrates the ways in which truth-telling is situated within asymmetrical relations of power.

The resolution of this scandal consolidates the dominant interpretation of these forms of death and injury as tragic (Zehfuss, 2007), albeit avoidable, accidents (Owens, 2003). By late October 2001, US Psychological Operations (PSYOPs) radio had begun broadcasting the following message in Afghanistan:

Attention noble Afghan people. As you know, the coalition countries have been air-dropping daily humanitarian rations for you. … The food inside the bags is Halal and very nutritional. In areas away from where food has been dropped, cluster bombs will also be dropped. … Of course in future cluster bombs will not be dropped in areas where food is air-dropped. However, we do not wish to see an innocent civilian mistake the bombs for food bags and take it away believing that it might contain food. (BBC News, 2001)

This corrective intervention was also repeated for a domestic audience, with the Pentagon announcing in early November 2001 that it was changing the colour of the HDRs from yellow to apricot. Even the choice of colour is significant. Responding to rumours that the colour of the HDRs was to be changed to blue, a defence spokesman stated of the consultation process: ‘We’re still evaluating and researching what the right colour should be. … We want to avoid offending any cultural or religious sensibilities’ (Rhem, 2001). Agonising over the colour of the containers of their generosity and compassion, which has been so unfortunately miscommunicated, this interpretation and resolution of ethical failure comes to present another opportunity to reaffirm, rather than contest, the ethical character of the coalition forces.

The purpose of this analysis is not to clarify and contest the moral character of particular actors. The argument presented here is not that coalition forces in Afghanistan were unconcerned with the ethical implications of their actions, although there are grounds to be cautious about the same accusation being levelled at the Taliban. Instead, the aim of this analysis is to demonstrate the socio-political function of this scandal. Far from disrupting the relationship between the martial and the ethical, the dominant interpretation of civilian casualties is to read them as a demonstration of the varying ethical character of the involved parties. The occurrence of civilian casualties therefore offers coalition forces an opportunity to reaffirm and clarify their ethical character in face of their other. Put bluntly, through this process of narration, certain dead bodies come to be held up as demonstrating the ethical character of the very actors that killed them. Through this reading, the coalition emerge as flawed yet well-intentioned humanitarians while the Taliban emerge as a deliberately cruel and inhumane force. In this sense, the scripting of ethical failure is involved in reproducing the very representational logics that legitimate and necessitate the conflict in Afghanistan. We should be troubled with how this interpretation of civilian casualties appears to call for *more* not *less* death and injury. Read as such, we see how scandal, as a form of operational negativity, revitalises the very social order that it appears to demonstrate to be failing. This is the success of ethical failure.

While this section has focused upon developing a reading of the *hyper*-visibility of this scandal, we can see traces of the discomforting remainders that remain *un*-visible and unaccounted for within the terms of its problematisation and resolution. Speaking at the announcement of the new colour, a defence spokesman remarked that ‘we called it salmon in the beginning. People said don’t eat salmon if that’s the colour. So they call it now apricot’ (Collins, 2003). Interestingly, in this attempt to reassert the distinction between instruments of compassion and harm, the latter continues to bleed into the former. This lingering indeterminacy, prompted by the reference to rotten fish, incites us to consider whether there might be something violent about the terms of our compassion, our life, our peace. Put simply, what this scandal fails to account for is the violence of the softer, more developmental practices that accompany the more spectacular and kinetic moments of liberal intervention. Problematising bombs and not bread, this scandal serves to reproduce a reductionist account of what violence is and where violence is located, thereby obscuring the subtle and complex contours of violence that define the mundane and everyday spaces of Afghanistan. It is these sites that are the focus of the next reading.

### A textbook scandal

There is no such thing as a neutral subject. (Foucault, 2003: 5)

Writing in December 2001 for *Newsweek* magazine, Babak Dehghanpisheh (2001, emphasis added) reported his experience of stumbling across textbooks from the time of the Soviet invasion that he argued ‘*blurr[ed] the line* between military and civilian education’. Operating as ‘Jihad manuals’, the article details how the textbooks featured ‘lessons on recognising the enemies of Islam and hand-drawn diagrams on assembling AK-47 rifles’. Consider, for example, this mathematics problem taken from a fourth-grade textbook that was still being used in 2001: ‘A Kalashnikov bullet travels at 800 meters per second. A mujahed has the forehead of a Russian in his sights 3,200 meters away. How many seconds will it take the bullet to hit the Russian’s forehead?’ Or the following: ‘A group of mujahedin kill 178 out of 3,560 enemy soldiers in battle. What percentage of the enemy have they killed?’

These are not isolated examples. Reflecting the changing enmities and prejudices of successive regimes, Dehghanpisheh shows how the violently anti-Soviet propaganda that was infused into the curriculum by the mujahedeen would later be supplemented by the Taliban’s ‘own brand of combative Islamic studies’. The article claims that textbooks in Afghanistan ‘have long served the dual purpose of basic education and political indoctrination’.

The first line-drawing manoeuvre is clearly apparent in the *Newsweek* article’s understanding of the transgressive dimensions of these textbooks. Described as radicalising generations of Afghan children, the textbooks are denounced for being a mere semblance of real education. As with the iconoclasts, this denouncement of the perceived perversion of the basic character and purpose of education relies upon and reaffirms the authenticity of an underlying regulative ideal. As such, the identification of transgression offers us a privileged insight into widespread assumptions about the apolitical and non-violent role of education. This is the law to which this scandal pays homage.

Education has proven to be one of the most sustainable justifications for the continued involvement of the international community in Afghanistan. Responding to the widespread indictment of the education system within Afghanistan, a central objective of Senator Joe Biden’s (2001: S10143) *Agenda for Afghanistan* was the creation of ‘secular schools … to break the stranglehold of radical religious seminaries that have polluted a generation of Afghan boys’. Indeed, the *Newsweek* article outlines the process of curriculum development, facilitated by the international community, in which ‘new books will focus on the subject being taught instead of political matters’ (Dehghanpisheh, 2001). Reduced to a logic of recrimination, in which a practice is denounced for not adhering to the norm, the *Newsweek* article gestures towards an emerging resolution through the development of a moderate and apolitical curriculum. Crucially, it is through this second line-drawing manoeuvre that the international community is positioned as a representative, custodian and guarantor of a depoliticised and universalised account of education.

As we saw in relation to civilian casualties, the othering of ethical failure is a crucial component of the interventionary narrative in Afghanistan. In the *Newsweek* article, the logic of problematisation and resolution functions to reproduce an imaginative geography in which difference expressed in spatial terms, between ‘us’ and ‘them’, is used to secure the legitimacy and necessity of the international interventions. Responsibility for the textbooks is ‘theirs’ and located over ‘there’, leaving ‘us’ and ‘here’ simultaneously both innocent and capable. This allows the international interventions to be framed as ‘bold, brave and powerful’ actions taken by compassionate guardians of humanitarian principles (Biden, 2001: S10141). As Ann Orford (1999: 681) has argued, these representational practices often serve ‘to obscure the extent to which the international has itself contributed to the humanitarian crisis that has emerged’. This point was to be powerfully demonstrated with the emergence of additional revelations about the origins of these textbooks.

On 16 March 2002, President George W. Bush announced that new textbooks supplied by the US would ‘teach tolerance and respect for human dignity, instead of indoctrinating students with fanaticism and bigotry’ (cited in CNN, 2002). On 23 March 2002, the *Washington Post* ran an article entitled ‘From U.S., the ABC’s of jihad: Violent Soviet-era textbooks complicate Afghan education efforts’ (Stephens and Ottaway, 2002). The article reveals that many of the textbooks denounced by Bush were, in fact, printed and distributed by the University of Nebraska at Omaha’s (UNO) Centre for Afghanistan Studies, with funding from the United States Agency for International Development (USAID).

Detailing how ‘the United States spent millions of dollars to supply Afghan schoolchildren with textbooks filled with violent images and militant Islamic teachings [as] part of covert attempts to spur resistance to the Soviet occupation’, the article notes that even the Taliban considered the teaching materials suitable for use. Featuring stories which taught students that ‘[i]f infidels invade, jihad is the obligation of every Muslim’, the UNO textbooks seem to reorder the logic of responsibility by demonstrating the US’s historic role in the promotion of the radicalisation processes that it was now attempting to eradicate. In this sense, the *Washington Post* article appears to disturb the spatial imaginary upon which the *Newsweek* article, and the war in Afghanistan more generally, is premised.

Seemingly consistent with its historic role in courageous acts of truth-telling, the *Washington Post* article appears to expose an uncomfortable legacy of complicities that disrupt the established terms and logics of the ‘war on terror’. However, as with the *Newsweek* article, the *Washington Post* article serves to reaffirm the legitimacy of international involvement in Afghanistan through the managed exposure of ethical failure. As has been shown, the *Newsweek* article performs this function through an imaginative geography that constructs difference in spatial terms, locating responsibility over ‘there’ in order to demonstrate the difference between ‘us’ and ‘them’. By contrast, the *Washington Post* article performs this function by containing responsibility in temporal terms, legitimising the intervention through the difference between ‘then’ and ‘now’. In spite of appearing to raise challenging questions about the role of the US in cultivating radicalisation in Afghanistan, the performative force of this article is ultimately to reassure the reader of the conduct of the very actors it appears to denounce.

This is most powerfully demonstrated by the conclusion of the *Washington Post* article, in which the journalists effectively resolve the scandal that they have exposed. Citing a USAID official responsible for revising the textbooks, the article ends by describing the decision of the US government to ‘reprint the old books [having] purg[ed] the violent references’. This purging process involved ‘hasty revisions to the printing plates [as] Afghan educators laboured night and day, scrambling to replace rough drawings of weapons with sketches of pomegranates and oranges’. Just as the colour of HDRs was changed from yellow to apricot, so images of guns are replaced with images of pomegranates. It is upon such tenuous movements that the spectacle of resolution is performed, and that the line between war and peace in Afghanistan is redrawn. In the final sentence of the *Washington Post* article, a USAID official assures us that through this purging, ‘We turned it from a wartime curriculum to a peacetime curriculum’. It is upon these terms that we can see how, ultimately, the message of the article is that there is no scandal, as the managed exposure of historic complicity ultimately comes to secure the legitimacy of interventions being conducted in the present.

What would happen, though, if we refused this logic of resolution? What if we were to explore and ask questions about what remains unaccounted for within these terms? Understanding the third line-drawing manoeuvre allows us to unsettle this process of resolution and containment, which, in this instance, opens up new avenues for tracing the complex contours of violence that define the more mundane spaces of contemporary warfare. Motivating the interrogation of this manoeuvre is the desire to cultivate the ethical potential of a lingering anxiety, which seeks to highlight and contest the fragility of moments of resolution. It is an anxiety that urges us to reflect upon what remains outside the perimeters of outrage and transgression that are defined by the terms of this scandal.

In both the *Newsweek* and *Washington Post* articles, the spectacle of resolution revolves around the process of purging the textbooks of violent imagery and text. This is taken to symbolise the transition from a violent, wartime curriculum to a non-violent, peacetime curriculum. However, such a resolution relies upon a highly reductionist account of what constitutes violence and where it resides. With outrage focused primarily on moments of more episodic, kinetic and spectacular moments of violence, wartime scandals often fail to account for the mundane, subtle and complex forms of violence that constitute the contours of the everyday spaces of Afghanistan. The terms of accountancy within this scandal, for example, render *un*-visible the epistemic and structural violences that define the softer, more humanitarian practices that form an increasingly crucial dimension of liberal war.

Specifically, this scandal serves to enable, excuse and obscure the violence of transforming the Afghan education system as part of wider efforts to reorganise the social, economic, cultural and political fabric of Afghan society. The violence of this radically political process of transforming the space of the social must be more than a discomforting remainder that haunts strictly delineated expressions of outrage. Focusing upon these remainders allows us to contest the teleologies that are implicit within the grammar of this scandal, which also define dominant cultural understandings of the liberal ways of war and peace, and that serve to reduce the politics of education in post-conflict societies to a largely technocratic and managerial task of capacity-building.

Paying attention to the remainders allows us to view education as a biopolitical technique involved in the management of populations. Informing this process is the simultaneous promotion of desirable ways of life and the eradication of undesirable ways of life. Such a dynamic is clearly visible in the new curriculum, which the Afghan Ministry of Education (2002: 11) describes as being designed to ‘strengthen the spirit of national unity, peace, women’s rights and the environment, and reflect awareness and motivation to enable the students to launch campaigns against war, and terrorism on all fronts worldwide’. The desire to promote particular forms of conduct, such as ‘healthy life styles and constructive attitudes’, is part of an attempt to discourage children away from those ways of life associated with anti-government forces: ‘war, terrorism, use of narcotics and other unhealthy habits’ (Ministry of Education, 2002: 10). In this sense, the new curriculum is not balanced and value-free, but remains, as with the scandalous textbooks, a decentred and adversarial expression of a series of normative preferences for a particular way of life. It is in this sense that we must understand that within Afghanistan, ‘there is no such thing as a neutral subject’ (Foucault, 2003: 5).

The purpose of this section is not to fully elaborate upon such a critique, but rather to demonstrate that such lines of inquiry remain outside the terms of problematisation established by this scandal, and subsequently that their socio-political implications are therefore unaccounted for. Tracing the third line-drawing manoeuvre allows us to bring these lingering remainders to the forefront of inquiries into the ethical and cultural imaginaries that define the character and discourse of contemporary war.

## Conclusion: Beyond a politics of recrimination

This article has focused on the interpretation and socio-political function of wartime scandals. It has presented both an opportunity and a warning: an opportunity in terms of exploring what scandals can tell us; and a warning in terms of understanding the performative force of what scandals are already telling us. Understood as a series of line-drawing manoeuvres, this article has outlined how scandals offer us a privileged insight into the character and reproduction of the normative architecture through which contemporary war is governed. This method for critically reading wartime scandals offers a number of important insights and opportunities for engaging with the ways in which contemporary wartime violences are enabled, excused and obscured.

It is important to understand that the argument presented in this article does not entail a rejection of the possibility or desirability of ethical arguments about war. This may appear to be the direction in which Baudrillard gestures. Baudrillard’s provocation that there is no scandal may seem to be a vague, inadequate and potentially conservative response to acts of violence that many feel motivated to respond to. Just as it has been argued that denunciation is in danger of being intimately involved in the reproduction of the very violences that it seeks to problematise, it could be argued that refusing to accept that particular acts are scandalous runs the risk of forming a silent complicity with them.

A commitment to normative politics does not, however, entail a choice between speaking out against violence or remaining silent. Put simply, *to speak or not to speak* is not the problem we find ourselves confronted with. Such a formulation proceeds from the assumption that scandals and recrimination are the only possible ways to articulate concern with particular acts. Understood as such, this article would appear to challenge this mode of critique, thereby eradicating or seriously limiting the possibility of expressing outrage at wartime violence. Only if we accept this premise are we confronted by the decision to speak or to remain silent. This article does not aim to restrict our ability to articulate outrage about particular acts of wartime violence. Scandals are not the only ways of narrating instances of death and injury in war. Other ways of speaking are possible and other stories can be told (Shepherd, 2006: 401). Realising this can only serve to expand, rather than limit, the vocabulary and possibilities of critical thought beyond a restrictive politics of recrimination.

The point of this article is that it is because, not in spite, of the ‘truth’ of these violences that we must interrogate the socio-political function of this way of speaking, of bearing witness, of *speaking truth to power*. This article does not therefore dismiss the importance of ethical arguments; instead, it attempts to demonstrate just how important they are. Taking ethical arguments seriously requires us to recognise that they are not detached from the violences that they reflect upon. Bearing witness is not without consequences. Troublingly, this article has demonstrated that the denunciation of moments of ethical failure may, in fact, reproduce the very practices that appear to be disturbed. Scandals may therefore secure the legitimacy and necessity of *more* not *less* violence. This spiralling and bewildering causality highlights the complexity and ambiguity of critiquing war. The challenge for critical inquiries into war is therefore how we can formulate ethical arguments about war that do not reproduce the conditions of possibility for the very practices that they seek to contest. The challenge is to imagine what outrage might look like when it is not expressed through the logic of scandal. Put simply, this article has explored and unpacked the logics and performative force of wartime scandals; the challenge now is to think about how we might speak beyond them.

Answering this question is beyond the scope of this article. The answer is, however, unlikely to come in the form of locating a pure space that is detached from complicity. Political engagement is always already part of the world. Rather than attempting to reclaim the romantic space of the revolutionary figure who speaks truth to power, perhaps we should continue to find strategies for negotiating a more complex and messy ground. From here, outrage remains necessary but is also alive to the limits, prejudices, preferences and omissions that it always, perhaps inevitably, articulates. An end to outrage is undesirable, but a genuine commitment to it must be uncomfortable.

## Notes

1. On the issue of the ethics of transparency, see the special issue on ‘Transparency’ of *Cultural Studies — Critical Methodologies* (Teurlings and Stauff, 2014).

2. There has been a growing interest in the ethics of truth-telling inspired by Foucault’s (2010, 2011) writing on the notion of *parrhēsia* (see Cadman, 2010; Death, 2016; Walters, 2014). Focused less upon the veracity of a given statement, Foucault’s interest in *parrhēsia* approaches truth-telling as an activity or a role. Specifically, *parrhēsia* involves the risky and courageous act of speaking uncomfortable truths, which thereby exposes the truth-teller to the undetermined consequences of their frank criticism. Situated within Foucault’s wider thinking about the ethics of counter-conduct, the hope of *parrhēsia* is that it will stimulate ‘collective self-examination’ (Death, 2016: 214) and ‘have a positive impact on the affairs of the community’ (Walters, 2014: 279). Foucault’s writing on the ethical impact of scandal is not limited to *parrhēsia*. Foucault also elaborates upon the notion of ‘scandalous behaviour’, which ‘call[s] into question collective habits and standards of decency’ (Davidson, 2009: xxxii). Baudrillard offers a more cautious analysis of the ethical potential and associated risks of truth-telling and scandal. For Baudrillard, the risks of truth-telling, if we are to speak in such terms, do not fall on the precarious subject, in terms of their exposure to the consequences of their fearless speech; instead, the riskiness of scandal must be understood in terms of its blunting of the limits and possibilities of critical thought. It is in this sense that scandals perform a more conservative role in re-energising particular standards of decency than that envisaged by Foucault.

3. That scandals so often serve to individualise and isolate transgression should raise questions about the innocence of the line-drawing manoeuvres through which scandals are rendered intelligible. While each scandal must be approached in terms of its specificity, we must be mindful that a limited number of symbolic scandals may offer the semblance of accountability, thereby hiding a more general and deliberate logic of impunity.

4. The notion of the *hyper*-visible draws upon Welland’s (2013: 887) understanding of the *un*-visible. For Welland, ‘[t]o state that something is un-visible as opposed to invisible is to make clear that it is not that it is not there, or cannot be made visible, but that it has been obscured through particular discourses or dominant stories’. Similarly, the use here of the notion of the hyper-visible rather than simply the visible is to affirm that scandals, as apparent moments of exposure and revelation, are always a product of particular regimes of narration and intelligibility.
